# TLR2 ligation induces the production of IL-23/IL-17 via IL-6, STAT3 and NF-kB pathway in patients with primary Sjogren's syndrome

**DOI:** 10.1186/ar3780

**Published:** 2012-03-14

**Authors:** Seung-Ki Kwok, Mi-La Cho, Yang-Mi Her, Hye-Joa Oh, Mi-Kyung Park, Seon-Yeong Lee, Yun Ju Woo, Ji Hyeon Ju, Kyung-Su Park, Ho-Youn Kim, Sung-Hwan Park

**Affiliations:** 1 Division of Rheumatology, Department of Internal Medicine, The Catholic University of Korea,505 Banpo-dong, Seocho-gu, Seoul 137-701, Korea; 2 The Rheumatism Research Center, Catholic Research Institute of Medical Science, The Catholic University of Korea, 505 Banpo-dong, Seocho-gu, Seoul 137-701l, Korea

## Abstract

**Introduction:**

The study was undertaken to investigate the interrelation of toll-like receptor (TLR) and interleukin (IL)-17 in the salivary glands of patients with primary Sjogren's syndrome (pSS) and to determine the role of TLR and IL-17 in the pathophysiology of pSS.

**Methods:**

The expressions of various TLRs, IL-17 and the cytokines involved in Th17 cell differentiation including IL-6, IL-23, tumor necrosis factor-alpha (TNF-α) and IL-1β were examined by immunohistochemistry in salivary glands of pSS patients. The IL-17 producing CD4+ T cells (Th17 cells) were examined by flow cytometry and confocal staining in peripheral mononuclear blood cells (PMBCs) and salivary glands of pSS patients. After PBMCs were treated with TLR specific ligands, the induction of IL-17 and IL-23 was determined using real-time PCR and ELISA. The signaling pathway that mediates the TLR2 stimulated production of IL-17 and IL-23 was investigated by using treatment with specific signaling inhibitors.

**Results:**

We showed that TLR2, TLR4, TLR6, IL-17 and the cytokines associated with Th17 cells were highly expressed in salivary glands of pSS patients but not in controls. The expressions of TLR2, TLR4 and TLR6 were observed in the infiltrating mononuclear cells and ductal epithelial cells, whereas IL-17 was mainly observed in infiltrating CD4+ T cells. The number of IL-17 producing CD4+ T cells was significantly higher in pSS patients both in PBMCs and minor salivary glands. The stimulation of TLR2, TLR4 and TLR6 additively induced the production of IL-17 and IL-23 from the PBMCs of pSS patients especially in the presence of TLR2 stimulation. IL-6, signal transducer and activator of transcription 3 (STAT3) and nuclear factor-kappaB (NF-kB) pathways were implicated in the TLR2 stimulated IL-17 and IL-23.

**Conclusions:**

Our data demonstrate that TLR2 ligation induces the production of IL-23/IL-17 via IL-6, STAT3 and NF-kB pathway in pSS. Therefore, therapeutic strategies that target TLR/IL-17 pathway might be strong candidates for treatment modalities of pSS.

## Introduction

Sjögren's syndrome (SS) is a relatively common autoimmune exocrinopathy that is associated with infiltration of lymphocytes and is characterized by gradually progressive lachrymal and salivary dysfunction [1,2]. The exocrine dysfunction can occur alone (primary SS, or pSS) or in the presence of other systemic autoimmune diseases like rheumatoid arthritis (RA), systemic lupus erythematosus (SLE), and systemic sclerosis (secondary SS). In addition to presenting the manifestations due to exocrine dysfunction, it can present a number of systemic manifestations such as arthralgia, thyroiditis, renal involvement, peripheral neuropathy, cutaneous vasculitis, and lymphoma [1]. Although research on immunopathology, genetics, and virus has been carried out over the past couple of decades to elucidate the pathogenesis of SS, the etiology remains unknown.

The Toll-like receptor (TLR) family is the prototype of pattern recognition receptors that sense diverse pathogen-associated molecular patterns (PAMPs), including lipids, lipoproteins, proteins, and nucleic acids [3,4]. Recognition of PAMPs by TLRs activates intracellular signaling pathways and this culminates in the induction of inflammatory cytokines, chemokines, and interferons and upregulation of co-stimulatory molecules. Thereby, the family of TLRs expressed on antigen-presenting cells such as dendritic cells (DCs) and macrophages play critical roles in the subsequent development of adaptive immune response as well as in the induction of innate immune response [4,5]. It has been known that TLR signaling could contribute to the initiation and progression of various autoimmune diseases such as RA, experimental autoimmune encephalitis, myocarditis, kidney disease, hepatitis, SLE, diabetes, and experimental autoimmune uveitis as well as aging [6-12]. In regard to the role of TLRs in SS, it was reported that certain types of TLRs are expressed in salivary gland tissue and salivary gland cell lines [13,14]. However, the pathogenic role of TLRs in SS remains to be determined.

A couple of decades ago, Mosmann and colleagues [15] proposed that CD4^+ ^T cells differentiate into two subsets with reciprocal functions and patterns of cytokine secretion, termed T helper 1 (Th1) and Th2. This classical paradigm was maintained until 2005, when a distinct lineage of proinflammatory Th cell subsets, termed Th17 cells, was identified [16,17]. Th17 cells are characterized by the production of a proinflammatory cytokine, interleukin-17 (IL-17, also known as IL-17A). It is well known that various cytokines like IL-6, transforming growth factor-beta (TGF-β), IL-23, and IL-1β contribute to the differentiation or amplification (or both) of Th17 cells [18,19]. Both IL-17 and Th17 cells are critically implicated in the pathogenesis of diverse autoimmune diseases [19-21], and it was reported that IL-17 or Th17 cells or both are highly expressed in the salivary glands of patients with SS [22-24]. Unfortunately, the exact pathophysiologic role of IL-17 in SS remains to be defined.

Given that both TLRs and IL-17 are upregulated in the salivary glands of patients with SS, we hypothesized that both TLRs and Th17-associated cytokines like IL-17, IL-23, and IL-6 are closely interrelated with each other and so are involved in the pathogenesis of SS. To address these issues, this study examined the expression of various TLRs and Th17-associated cytokines in the salivary glands of patients with SS. We evaluated the frequency of IL-17-producing CD4^+ ^T cells in both peripheral blood mononuclear cells (PBMCs) and minor salivary glands. Using PBMCs of patients with SS, we also investigated whether the stimulation of TLRs induces the production of Th17-associated cytokines. Finally, we uncovered the probable signaling pathway that mediated the TLR-stimulated production of Th17-associated cytokines.

## Materials and methods

### Patients

Forty patients (3 males and 37 females), all of whom fulfilled the SS classification criteria proposed by the American-European Consensus Group [25], and 20 healthy controls matched for age and sex were included in the study. Five disease control subjects presented with sicca symptoms to a rheumatology clinic, but neither autoantibody (anti-Ro or anti-La) nor results of labial salivary gland biopsy fulfilled the classification criteria for SS. All of the patients had pSS. All the subjects gave informed consent before the study. The study received the approval of the institutional review board of Seoul St. Mary's Hospital.

### Labial salivary gland biopsy

Labial minor salivary gland biopsies were obtained with informed consent from 21 patients who underwent diagnostic evaluation of sicca symptoms indicative of SS. Among the biopsies, 16 were diagnosed as pSS and the other five were defined as disease controls. Five or six minor salivary gland lobules were carefully harvested and placed into formalin fixative. Standard paraffin preparations were prepared and these were sectioned at 5-μm thickness and then stained with hematoxylin and eosin. The slides were examined for the presence of lymphocytic infiltrates or foci or both by three observers (using standardized criteria). A 'focus' was defined as an aggregate of at least 50 lymphocytes with a few plasma cells. The focus score was reported as the number of foci per 4 mm^2 ^of tissue [26].

### Clinical and laboratory profiles

All of the patients underwent extensive serologic evaluations, which included tests for the presence of antinuclear antibodies, anti-SSA/Ro, anti-SSB/La, anti-double-stranded DNA, and rheumatoid factor as well as erythrocyte sedimentation rate (ESR), and the levels of globulin and the subtypes of immunoglobulin. In addition, all of the patients underwent an extensive medical examination.

### Immunohistochemical staining of TLR2, TLR4, TLR6, IL-17, IL-23, STAT3, phospho-STAT3(s727), p-IKB, IL-6, TNF-α, and IL-1β

The paraffin-embedded slides were deparaffinized by immersion in xylene, followed by dehydration in ethanol. The endogenous peroxidase activity was blocked by 3% hydrogen peroxide. The sections were incubated for 30 minutes at room temperature with blocking solution containing normal sera and avidin block (Vector Laboratories, Burlingame, CA, USA). The tissue sections were incubated overnight at 4°C with primary antibodies directed against TLR2, TLR4, TLR6, and IL-1β (Santa Cruz Biotechnology, Inc., Santa Cruz, CA, USA) and IL-17, IL-23, and TNF-α (R&D Systems, Inc., Minneapolis, MN, USA) and signal transducer and activator of transcription 3 (STAT3), phospho-STAT3 (S727), and p-IKB (Cell Signaling Technology, Inc., Beverly, MA, USA), and IL-6 (Abcam, Cambridge, UK). Isotype controls were done with goat IgG or rabbit IgG. The slides were washed for 5 minutes, followed by a 20-minute incubation with biotinylated secondary antibodies (Vector Laboratories). After a 15-minute wash, slides were incubated for 1 hour with horseradish peroxidase conjugated with avidin by using the Vecterstain ABC Elite (Vector Laboratories). The staining was developed by using diaminobenzidine substrate (Dako, Carpinteria, CA, USA), and counterstaining was performed with hematoxylin. Samples were photographed with a photomicroscope (Olympus, Tokyo, Japan).

### Confocal microscope

Cryosections (7 μm thick) were fixed with acetone, blocked with 10% goat serum, and stained with anti-CD4-PerCP-Cy5.5 and anti-IL-17 fluorescein isothiocyanate (eBioscience, San Diego, CA, USA). Fluorescence images were acquired by using an LSM 510 confocal microscope (Carl Zeiss, Jena, Germany).

### Isolation and culture of the mononuclear cells

Heparinized peripheral blood (20 mL) was aseptically collected from the patients with SS and healthy controls. PBMCs were isolated by density gradient centrifugation on Ficoll-Hypaque. The cells were then resuspended in RPMI 1640 medium supplemented with 10% fetal bovine serum (Gibco-BRL, now part of Invitrogen Corporation, Carlsbad, CA, USA), 100 U/mL penicillin, 100 μg/mL streptomycin, and 2 mM L-glutamine. Each culture was performed in triplicate at a density of 1 × 10^6 ^cells per well in 24 wells. The cells were incubated with peptidoglycan, lipopolysaccharide, and zymosan A (Sigma-Aldrich, St. Louis, MO, USA) for the indicated times. The cells were incubated for the indicated times.

### Cytokine enzyme-linked immunosorbent assay

The concentrations of IL-17, IL-23, TNF-α, IL-6, and IL-1β (R&D Systems, Inc.) in the supernatants of the cultured cells were measured by enzyme-linked immunosorbent assay (ELISA) in accordance with the instructions of the manufacturer. In some experiments, neutralizing antibodies were added to cell culture and then the cytokines (IL-23 and IL-6) were measured. Neutralizing antibodies do not have an effect on their measurement in the same culture supernatants.

### Detection of intracellular IL-17 by flow cytometry

Cell pellets were prepared from PBMCs of healthy controls and patients with SS. PBMCs were stimulated with 25 ng/mL poly(methyl acrylate), 250 ng/mL ionomycin (Sigma-Aldrich), and Golgi stop (BD Pharmingen, San Diego, CA, USA) for 4 hours. To examine the population of IL-17^+ ^T cells, cells were stained with anti-human CD4-PEcy5.5 monoclonal antibody (mAb) (BD Pharmingen) and anti-human CD8-APC mAb (BD Pharmingen) for 30 minutes at 4°C. Cells were permeabilized and fixed with CytoPerm/CytoFix (BD Pharmingen) in accordance with the instructions of the manufacturer, stained further with anti-human IL-17-PE mAb (BD Pharmingen), and subjected to flow cytometric analysis (FACSCalibur; BD Biosciences).

### Real-time PCR with SYBR green and reverse transcriptase-PCR

mRNA was extracted by using RNAzol B (BioTex Labs, San Antonio, TX, USA) in accordance with the instructions of the manufacturer. Reverse transcription of 2 μg of total mRNA was conducted at 42°C by using the Superscript Reverse Transcription system (Takara, Shiga, Japan). Real-time polymerase chain reaction (PCR) amplification of cDNA aliquots was performed by adding SYBR green I (Roche Diagnostics, Mannheim, Germany), and human IL-23, IL-17, and β-actin were amplified for IL-23 p19 by using the sense primer 5'-GCA GAT TCC AAG CCT CAG TC-3' and the anti-sense primer 5'-TTC AAC ATA TGC AGG TCC CA-3', for IL-17 by using the sense primer 5'-CAA CCG ATC CAC CTC ACC TT-3' and the anti-sense primer 5'-GGC ACT TTG CCT CCC AGA T-3', and for β-actin by using the sense primer 5'-GGA CTT CGA GCA AGA GAT GG-3' and the anti-sense primer 5'-TGT GTT GGG GTA CAG GTC TTT G-3' in a LightCycler™ (Roche Diagnostics). The relative expression levels were calculated by normalizing the IL-23 and IL-17 levels to the endogenously expressed housekeeping gene (β-actin). Melting-curve analysis was performed immediately after the amplification protocol under the following conditions: 0 seconds (hold time) at 95°C, 15 seconds at 65°C, and 0 seconds (hold time) at 95°C. The temperature change rate was 20°C/second except in the final step, when it was 0.1°C/second. The crossing point (*C*_p_) was defined as the maximum of the second derivative from the fluorescence curve.

Reverse transcriptase-PCR amplification of cDNA aliquots was performed by adding 2.5 μM dNTPs and 2.5 U Taq DNA polymerase (Takara), and human TLR2, TLR4, and TLR6 were amplified for TLR2 by using the sense primer 5'-GCC AAA GTC TTG ATT GAT TGG-3' and the anti-sense primer 5'-TTG AAG TTC TCC AGC TCC TG-3', for TLR4 by using the sense primer 5'-TGG ATA CGT TTC CTT ATA AG-3' and the anti-sense primer 5'-GAA ATG GAG GCA CCC CTT C-3', and for TLR6 by using the sense primer 5'-TAG GTC TCA TGA CGA AGG AT-3' and the anti-sense primer 5'-GGC CAC TGC AAA TAA CTC CG-3'. Reactions were processed in a DNA thermal cycler (PerkinElmer Cetus, Wellesley, MA, USA) through 30 cycles at 95°C for 30 seconds, 60°C for 30 seconds, and 72°C for 30 seconds for TLR2, TLR4, and TLR6.

### Statistical analysis

Statistical analyses were performed by using SAS software (version 9; SAS Institute Inc., Cary, NC, USA). The experimental values are presented as mean ± standard deviation. Differences in the TLR expressions in the minor salivary glands of various groups were analyzed by using the Kruskal-Wallis test. Comparisons of numerical data between two groups were performed by the Student *t *test or Mann-Whitney *U *test. *P *values of less than 0.05 were considered statistically significant.

## Results

### TLR2, TLR4, TLR6, IL-17, and IL-23 are highly expressed in patients with SS

**Figure 1:**
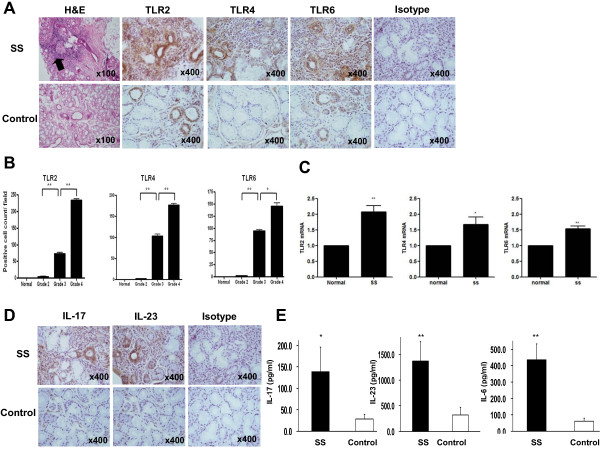
**Expressions of TLR2, TLR4, TLR6, IL-17, and IL-23 in minor salivary glands of patients with SS and disease controls**. **(a) **The expressions of TLR2, TLR4, and TLR6 in the minor salivary glands of patients with SS (upper panel) and disease controls who have sicca symptoms but do not fulfill the classification criteria for SS (lower panel). Hematoxylin and eosin (H&E) staining showed marked infiltration of inflammatory cells in salivary gland tissue of patients with SS (arrow in upper panel). Immunostaining was performed by using specific antibodies in patients with SS (*n *= 16) and disease controls (*n *= 5). Cells stained with each antibody are shown in brown. Intense staining of TLR2, TLR4, and TLR6 is observed in the ductal epithelial cells and infiltrating mononuclear cells in patients with SS, whereas isotype control did not show any immunoreactivity. **(b) **The labial salivary gland specimens were divided according to the grade (approximately 0 to 4) of lymphocytic infiltration (see Materials and methods). The cells showing positive staining of TLRs were enumerated visually at higher magnification (projected on a screen) by four individuals, and the mean values are presented in the form of a histogram. *P < 0.05, **P < 0.01. **(c) **Peripheral blood mononuclear cells were isolated from patients with SS (*n *= 5) and healthy controls (*n *= 5). The expressions of TLR2, TLR4, and TLR6 mRNA were evaluated by reverse transcription-polymerase chain reaction. *P < 0.05, **P < 0.01 compared with healthy controls. **(d) **IL-17 and IL-23 are highly expressed in the infiltrating mononuclear cells. Ductal epithelial cells are variably positive for IL-17 and IL-23. Expressions of IL-17 and IL-23 in the disease controls are quite weak in comparison with that in patients with SS. **(e) **Serum levels of IL-17, IL-23, and IL-6 were determined by enzyme-linked immunosorbent assay in patients with SS (*n *= 40) and healthy controls (*n *= 20). *P < 0.05, **P < 0.01 compared with healthy controls. IL, interleukin; SS, Sjögren's syndrome; TLR, Toll-like receptor.

To determine the roles of TLRs and the Th17-associated cytokines in the pathogenesis of SS, we first examined the *in situ *expression of various TLRs (TLR2, TLR4, and TLR6) and Th17-associated cytokines such as IL-17 and IL-23 by immunohistochemistry in the minor salivary glands of 16 patients with SS and five disease controls. The expressions of TLR2, TLR4, and TLR6 were significantly higher in patients with SS than in disease controls (Figure 1a). Interestingly, as the focus score (grade) increased, the expression degree of TLR2, TLR4, and TLR6 increased (Figure 1b). The mRNA expressions of TLR2, TLR4, and TLR6 were also significantly higher in PBMCs of patients with SS than in healthy controls (Figure 1c). As shown in Figure 1d, the expressions of IL-17 and IL-23 were also significantly higher in the minor salivary glands of patients with SS, as was noted in a previous report [23]. Our immunohistochemical analysis showed that intense staining of TLR2, TLR4, and TLR6 was evident in infiltrating mononuclear cells and ductal epithelial cells. IL-17 and IL-23 were highly expressed mainly in the infiltrating mononuclear cells. All of the biopsy specimens showed similar staining patterns. We also checked the serum levels of IL-17, IL-23, and IL-6 in the patients with SS and the healthy controls. The result showed that the serum levels of IL-17, IL-23, and IL-6 were significantly higher in patients with SS than in healthy controls (Figure 1e).

### IL-17-producing CD4^+ ^T cells (Th17 cells) are increased in patients with SS

**Figure 2:**
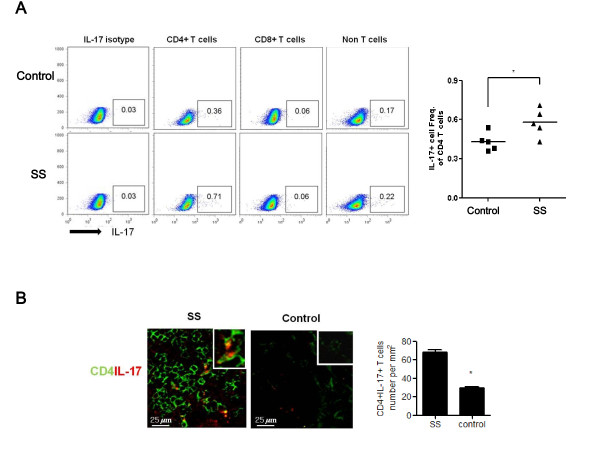
**IL-17-producing CD4^+ ^T cells in PBMCs and minor salivary glands of patients with SS and controls**. **(a) **After the isolation of PBMCs from healthy controls (*n *= 5) and patients with SS (*n *= 5), the populations of IL-17-producing CD4^+ ^T cells, CD8^+ ^T cells, and non-T cells were analyzed by using antibodies specific for CD4, CD8, and IL-17 by intracellular flow cytometric analysis. The representative figure is shown in the left panel. The percentage of IL-17-producing CD4^+ ^T cells is shown in the right panel. *P < 0.05, compared with healthy controls. **(b) **Salivary gland tissues from patients with SS or disease controls were stained for IL-17-producing CD4^+ ^T cells by using antibodies specific for IL-17 (green) and CD4 (red). The population of IL-17-producing CD4^+ ^T cells was analyzed by using laser confocal microscopy (400×). Cells showing positive IL-17-producing CD4^+ ^T cells staining were enumerated visually at higher magnification (projected on a screen) by four individuals, and the mean values are presented in the form of a histogram. *P < 0.05, compared with disease controls. IL, interleukin; PBMC, peripheral blood mononuclear cell; SS, Sjögren's syndrome.

We determined which cell populations are major sources of IL-17 in patients with SS by using PBMCs by intracellular flow cytometric analysis. As shown in Figure 2a, the major IL-17-producing cells were CD4^+ ^T cells in both patients with SS (*n *= 5) and healthy controls (*n *= 5). The percentage of IL-17-producing CD4^+ ^T cells (Th17 cells) was significantly higher in patients with SS than in healthy controls. However, there were no differences in IL-17-producing CD8^+ ^T cells or IL-17-producing non-T cells between the groups (data not shown). Using confocal microscopy, we also detected that the number of IL-17-producing CD4^+ ^T cells was significantly higher in minor salivary glands of patients with SS than in disease controls (Figure 2b).

### The stimulation of TLR2, TLR4, and TLR6 promotes the production of IL-17 and IL-23 in patients with SS

**Figure 3:**
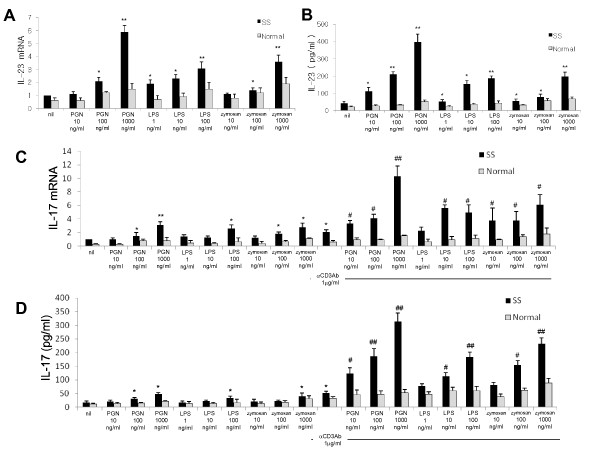
**The stimulation of TLR2, TLR4, and TLR6 upregulates the production of IL-17 and IL-23 in PBMCs from patients with SS**. **(a) **IL-23 mRNA expression induced by TLR-specific ligands. PBMCs from patients with SS (*n *= 5) and healthy controls matched for age and sex (*n *= 5) were cultured with ligands for TLR2, TLR4, and TLR6 for 12 hours. The expression of IL-23 mRNA was evaluated by real-time PCR. *P < 0.05, **P < 0.01 compared with nil. **(b) **IL-23 production induced by TLR-specific ligands. PBMCs from patients with SS and healthy controls were cultured with ligands for TLR2, TLR4, and TLR6 for 48 hours. The concentration of IL-23 in the culture supernatants was measured. *P < 0.05, **P < 0.01 compared with nil. **(c) **IL-17 mRNA expression induced by TLR-specific ligands. PBMCs from patients with SS and healthy controls were cultured with ligands for TLR2, TLR4, and TLR6 in the presence or absence of anti-CD3 for 12 hours. The expression of IL-17 mRNA was evaluated by real-time PCR. *P < 0.05, **P < 0.01 compared with nil. ^#^*P *< 0.05, ^##^*P *< 0.01 compared with anti-CD3-treated cells. **(d) **IL-17 production induced by TLR-specific ligands. PBMCs from patients with SS and healthy controls were cultured with ligands for TLR2, TLR4, and TLR6 in the presence or absence of anti-CD3 for 48 hours. The concentration of IL-17 in the culture supernatants was measured. *P < 0.05, **P < 0.01 compared with nil. ^#^*P *< 0.05, ^##^*P *< 0.01 compared with anti-CD3-treated cells. IL, interleukin; LPS, lipopolysaccharide; PBMC, peripheral blood mononuclear cell; PCR, polymerase chain reaction; PGN, peptidoglycan; SS, Sjögren's syndrome; TLR, Toll-like receptor.

We investigated the expression and production of IL-23 in the PBMCs obtained from the patients with SS and the healthy controls after stimulation of TLRs by their specific ligands. To characterize the response to TLR stimulation, the concentration of IL-23 in the culture supernatant was measured by ELISA and the mRNA production of IL-23 was assessed by real-time PCR. As shown in Figure 3a, the production of IL-23 mRNA markedly increased after the stimulation of TLR2, TLR4, and TLR6 and the production of IL-23 mRNA was significantly higher in patients with SS than in healthy controls. The concentration of IL-23 in the culture supernatant was markedly increased in the presence of the stimulation of TLR2, TLR4, and TLR6 in patients with SS (Figure 3b). The production and concentration of IL-23 increased in a dose-dependent manner, especially in the patients with SS.

We also checked the production and concentration of IL-17 in the PBMCs obtained from the patients with SS and healthy controls after stimulation of TLRs by their specific ligands. The results showed that the production and concentration of IL-17 markedly increased in the presence of anti-CD3 stimulation in the patients with SS but not in the healthy controls (Figure 3c, d).

### The stimulation of TLR2, TLR4, and TLR6 additively induces the production of IL-23 and IL-17 in patients with SS

**Figure 4:**
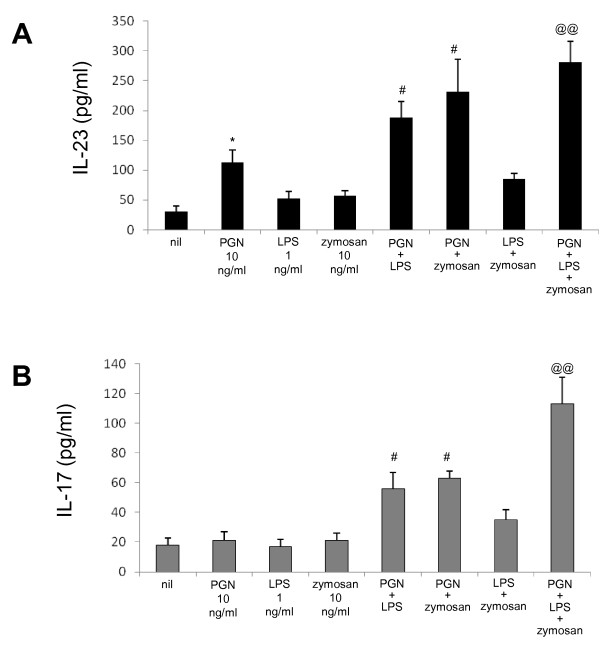
**The additive effect of TLR2, TLR4, and TLR6 ligation on the production of IL-17 and IL-23**. IL-23 **(a) **and IL-17 **(b) **production stimulated additively by TLR2, TLR4, and TLR6 ligation. Peripheral blood mononuclear cells from patients with Sjögren's syndrome (*n *= 5) were cultured with ligands for TLR2 and/or TLR4 and/or TLR6 for 48 hours. The concentrations of IL-23 and IL-17 in the culture supernatants were measured by sandwich enzyme-linked immunosorbent assay. Data are expressed as the mean ± standard deviation. *P < 0.05 compared with nil. ^#^*P *< 0.05 compared with TLR2 ligand-treated cells. ^@@^*P *< 0.01 compared with TLR2, TLR4, and TLR6 ligand-treated cells. IL, interleukin; LPS, lipopolysaccharide; PGN, peptidoglycan; TLR, Toll-like receptor.

We examined the PBMCs from the patients with SS to determine whether there were additive or synergistic effects of various TLR-specific ligands on the production of IL-23 and IL-17. We found that the stimulatory impact of TLR2 ligation on IL-23 and IL-17 production was augmented by TLR4 or TLR6 ligation or both. However, in the absence of TLR2 stimulation, there was no additive or synergistic effect of TLR4 and TLR6 stimulation on the production of IL-23 and IL-17 (Figure 4a, b).

### The production of IL-23 and IL-17 induced by TLR2 stimulation is mediated by IL-6, STAT3, and NF-κB pathways in patients with SS

**Figure 5:**
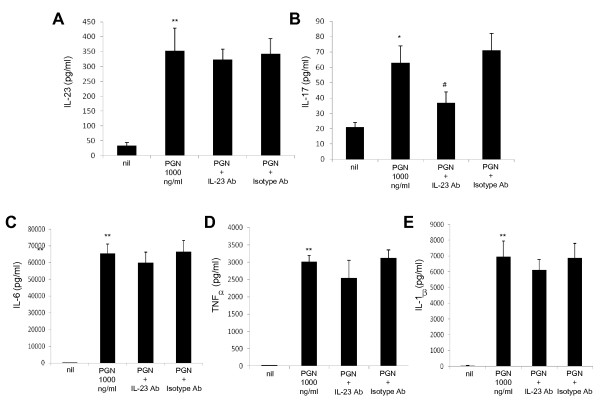
**Blocking of stimulatory effect of TLR2 ligation on IL-17 production by anti-IL-23 antibody**. The productions of IL-23 **(a)**, IL-17 **(b)**, IL-6 **(c)**, TNF-α **(d)**, and IL-1β **(e) **induced by TLR2 ligand in the presence or absence of neutralizing antibody (NE) of IL-23. Peripheral blood mononuclear cells from patients with Sjögren's syndrome (*n *= 5) were cultured with TLR2 ligand (PGN) in the presence or absence of neutralizing antibody (NE) of IL-23 for 48 hours. The concentrations of IL-23, IL-17, IL-6, TNF-α, and IL-1β in the culture supernatants were measured by sandwich enzyme-linked immunosorbent assay. Data are expressed as the mean ± standard deviation. *P < 0.05, **P < 0.01 compared with nil. ^#^*P *< 0.05 compared with PGN-treated cells. Ab, antibody; IL, interleukin; NE, neutralizing antibody; PGN, peptidoglycan; TLR, Toll-like receptor; TNF-α, tumor necrosis factor-alpha.

**Figure 6:**
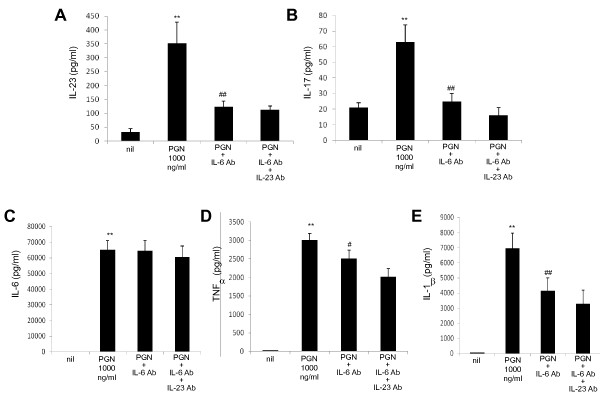
**Blocking of stimulatory effect of TLR2 ligation on IL-23, IL-17, TNF-α, and IL-1β production by anti-IL-6 antibody**. The production of IL-23 **(a)**, IL-17 **(b)**, IL-6 **(c)**, TNF-α **(d)**, and IL-1β **(e) **induced by TLR2 ligand in the presence or absence of neutralizing antibodies (NE) of IL-6 or IL-23 or both. Peripheral blood mononuclear cells from patients with Sjögren's syndrome (*n *= 5) were cultured with TLR2 ligand (PGN) in the presence or absence of neutralizing antibodies (NE) of IL-6 or IL-23 or both for 48 hours. The concentration of IL-23, IL-17, IL-6, TNF-α, and IL-1β in the culture supernatants were measured by sandwich enzyme-linked immunosorbent assay. Data are expressed as the mean ± standard deviation. **P < 0.01 compared with nil. ^#^*P *< 0.05, ^##^*P *< 0.01 compared with PGN-treated cells. Ab, antibody; IL, interleukin; NE, neutralizing antibody; PGN, peptidoglycan; TLR, Toll-like receptor; TNF-α, tumor necrosis factor-alpha.

To elucidate the signaling pathway by which the TLR2-stimulated production of IL-23/IL-17 is mediated, we first examined whether IL-23, a major causative factor for Th17 cell amplification, is implicated in the IL-17 production induced by TLR2 stimulation by using PBMCs from the patients with SS. As shown in Figure 5b, treatment with anti-IL-23 antibody blocked the stimulatory effect of TLR2 ligation on IL-17 production. However, anti-IL-23 treatment did not have an effect on the production of other cytokines like IL-23, IL-6, TNF-α, and IL-1β (Figure 5a, c-e. Next, we investigated whether IL-6, a major causative factor for Th17 cell differentiation, is involved in the IL-17 production induced by TLR2 ligation. Interestingly, treatment with anti-IL-6 antibody blocked the stimulatory effect of TLR2 ligation on the production of IL-23, TNF-α, and IL-1β as well as IL-17 (Figure 6a, b, d, e), and additional treatment with anti-IL-23 antibody did not have a meaningful impact. Anti-IL-6 antibody treatment did not affect the production of IL-6 (Figure 6c). These findings suggest that TLR2-stimulated production of IL-17 and IL-23 is mediated by IL-6.

**Figure 7:**
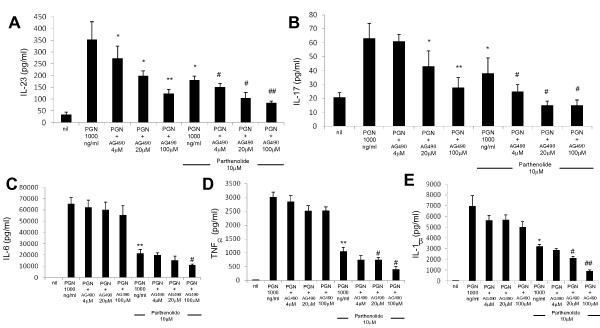
**Inhibition of STAT3 or NF-κB signaling or both reverses the production of IL-23, IL-17, IL-6, TNF-α, and IL-1β stimulated by TLR2 ligation**. The production of IL-23 **(a)**, IL-17 **(b)**, IL-6 **(c)**, TNF-α **(d)**, and IL-1β **(e) **induced by TLR2 ligand in the presence or absence of inhibitor of STAT3 or NF-κB or both. Peripheral blood mononuclear cells from patients with SS (*n *= 5) were cultured with TLR2 ligand (PGN) in the presence or absence of STAT3 inhibitor (AG490 4, 20, and 100 μM) or NF-κB inhibitor (parthenolide 10 μM) or both for 48 hours. The concentrations of IL-23, IL-17, IL-6, TNF-α, and IL-1β in the culture supernatants were measured by sandwich enzyme-linked immunosorbent assay. Data are expressed as the mean ± standard deviation. *P < 0.05, **P < 0.01 compared with PGN-treated cells. ^#^*P *< 0.05, ^##^*P *< 0.01 compared with PGN- and NF-κB inhibitor-treated cells. IL, interleukin; NF-κB, nuclear factor-kappa-B; PGN, peptidoglycan; SS, Sjögren's syndrome; STAT, signal transducer and activator of transcription; TLR, Toll-like receptor; TNF-α, tumor necrosis factor-alpha.

It is known that STAT3 appears to be the most important for IL-6 signaling [27] and that nuclear factor-kappa-B (NF-κB) is the most important for TLR signaling [3,5]. Considering this finding, we investigated the intracellular signaling pathway that mediated TLR2-stimulated production of IL-23/IL-17. As expected, treatment with STAT3 inhibitor (AG490) blocked the stimulatory effect of TLR2 ligation on the production of IL-23 and IL-17 (Figure 7a, b). Surprisingly, treatment with NF-κB inhibitor (parthenolide) showed that the TLR2-stimulated production of IL-6, TNF-α, and IL-1β as well as IL-23 and IL-17 was blocked. Moreover, simultaneous inhibition of both STAT3 and NF-κB showed additive inhibition of TLR2-stimulated production of the Th17-associated cytokines (Figure 7a-e).

**Figure 8:**
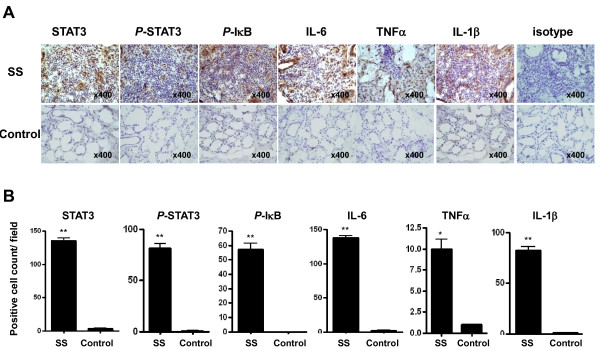
**Increased expressions of STAT3, *P*-STAT3, IκB, IL-6, IL-1β, and TNF-α in minor salivary glands of patients with SS**. **(a) **The expressions of STAT3, phospho-STAT3 (*P*-STAT3), phosphor-IκB (*P*-IκB), IL-6, TNF-α, and IL-1β in minor salivary glands of patients with SS (upper panel) and disease controls (lower panel). Immunostaining was performed by using specific antibodies in patients with SS (*n *= 16) and disease controls (*n *= 5). Cells stained with each antibody are shown in brown. Intense stainings of STAT3, *P*-STAT3, *P*-IκB, IL-6, TNF-α, and IL-1β are observed in the infiltrating mononuclear cells in patients with SS, whereas isotype control did not show any immunoreactivity. Expressions of STAT3, *P*-STAT3, *P*-IκB, IL-6, IL-1β, and TNF-α in the disease controls are quite weak in comparison with those in patients with SS. Representative results are shown. **(b) **The cells showing positive staining were enumerated visually at higher magnification (projected on a screen) by four individuals, and the mean values are presented in the form of a histogram. *P < 0.05, **P < 0.01 compared with disease controls. IL, interleukin; *P*-IκB, phospho-IκB; SS, Sjögren's syndrome; STAT, signal transducer and activator of transcription; TNF-α, tumor necrosis factor-alpha.

Finally, to verify that the above observations are meaningful in patients with SS, we examined the expression of STAT3, phospho-STAT3, phosphor-IκB, IL-6, TNF-α, and IL-1β in the minor salivary glands of patients with SS by immunohistochemistry. As shown in Figure 8a and 8b, the expressions of STAT3, phospho-STAT3, and phosphor-IκB were significantly higher in patients with SS than in the disease controls. In addition, the cytokines that are implicated in Th17 cell differentiation, like IL-6, TNF-α, and IL-1β, are highly expressed in the salivary glands of the patients with SS in comparison with the disease controls. Collectively, these findings suggest that TLR2 ligation induces the production of IL-23/IL-17 via the IL-6, STAT3, and NF-κB pathways.

## Discussion

SS is a chronic autoimmune disease of the exocrine glands and is characterized by an infiltration of lymphocytes and a female predominance. Although the pathogenesis of SS remains to be determined, the pathologic hallmark is an extensive lymphocytic infiltration of the salivary glands [1]. The majority of infiltrating cells are T cells, and approximately 60% to 70% of the infiltrating T cells bear the CD4 phenotype [28], suggesting that CD4^+ ^T cells contribute to the pathophysiology of SS [29].

Among CD4^+ ^T-cell subsets, Th17 cells are a unique CD4^+ ^T-cell subset and are characterized by production of IL-17. IL-17 is a highly inflammatory cytokine with robust effects on stromal cells, resulting in the production of inflammatory cytokines and recruitment of leukocytes, and this creates a link between innate and adaptive immunity [19]. It is well known that Th17 cells and IL-17 play an important role in the pathogenesis of a diverse group of immune-mediated diseases, including psoriasis [30,31], RA [32,33], multiple sclerosis [34], inflammatory bowel disease [35], and asthma [36]. In regard to the study of IL-17 in SS, previous studies support the finding that IL-17 or Th17 cells or both are upregulated in the salivary glands of patients with SS [22,24]. However, the pathophysiologic role of IL-17 is still undetermined.

As mentioned in the Introduction, TLRs, leading players in adaptive immunity as well as in innate immunity, have been thought to play a role in the pathogenesis of various human autoimmune inflammatory diseases. In addition, it has been reported that the expression of TLRs is upregulated in the salivary glands of patients with SS [13,14]. Thus, it can be speculated that increased TLRs and IL-17/Th17 cells in salivary glands might closely interact and so contribute to the pathogenesis of SS.

There have been discordant results in regard to the role of TLR stimulation in Th17 cell differentiation in humans and mice. Like us, Aliahmadi and colleagues [37] demonstrated that TLR2 activation promotes human Th17 polarization. Yu and colleagues [38] showed that human plasmacytoid DCs support Th17 cell effector function in response to TLR7 ligation. In contrast, Loures and colleagues [39] reported that TLR2 is a negative regulator of Th17 cells in mice.

In this study, we demonstrated that the expressions of TLR2, TLR4, and TLR6 were increased in the salivary glands of patients with SS in comparison with the disease controls. Moreover, not only IL-17 but IL-6 and IL-23, the major promoting factors in Th17 differentiation and amplification, were highly expressed. Using PBMCs from patients with SS, we also showed that stimulation of TLR2, TLR4, and TLR6 with specific ligands additively promoted the production of IL-17 and IL-23 in the presence of TLR2 stimulation, thus verifying that the increased TLRs and IL-17 in the salivary glands of patients with SS are biologically meaningful. Eventually, we investigated the signaling pathway by which TLR2 stimulation induces the production of IL-17 and IL-23 and we demonstrated that the IL-6, STAT3, and NF-κB pathways are implicated in TLR2-stimulated production of IL-23 and IL-17.

It is known that glandular epithelial cells appear to have the central role in the induction and perpetuation of tissue inflammatory reactions in patients with pSS [40,41] and have an increased rate of apoptosis [42]. Given the central role of glandular epithelial cells in local immune response and the immunohistochemical results presented in our study showing variably positive staining of ductal epithelial cells for IL-17 and IL-23 (Figure 1d), the role of epithelial cells in directing local immune responses could be more direct. Further investigations are needed to clarify the issue.

There have been a few previous reports that have tried to verify the role of Th17 cells and its associated cytokines like IL-17, IL-23, and IL-6 in patients with SS [22-24]. Like our report, these reports demonstrated that the expressions of Th17-associated cytokines in salivary glands are significantly higher in patients with SS than in controls (Figures 1d and 8a). However, conflicting results exist when it comes to the circulating levels of Th17-associated cytokines like IL-17, IL-23, and IL-6 in patients with SS. Like us, Katsifis and colleagues [22] showed that the serum levels of IL-17, IL-23, and IL-6 are significantly higher in patients with SS (Figure 1e). The authors also showed that serum levels of IL-6 and IL-23 are positively correlated with ESR in patients with SS [22]. In contrast, Nguyen and colleagues [23] reported that there were no differences in the serum levels of IL-17 and IL-6 between patients with SS and controls, although the IL-6 levels in the saliva from patients with SS exhibited nearly a threefold increase over those in the saliva from controls subjects. Studies that include a large number of patients will be required to clarify this discrepancy between the different studies.

The association between the IL-17 expression and immunopathologic features has been described [22,23,43]. One previous report showed that IL-17^+ ^mononuclear cell infiltrations in salivary glands progressively increased with higher biopsy focus score (*P *< 0.0001) and that the IL-17 mRNA expression of whole salivary gland positively correlated with ESR levels [22]. The report also showed that TGF-β, IL-6, and IL-23, which are the requisite promoters of Th17 differentiation, were found in abundance in the salivary glands of patients with SS, thus demonstrating that the microenvironment of salivary glands in patients with SS is full of factors that are known to foster local Th17 lineage polarization [22]. In our study, not only IL-17 but also IL-23, IL-6, IL-1β, and TNF-α, which are known to promote Th17 differentiation and amplification [19], were highly expressed in the salivary glands of patients with pSS in comparison with the disease controls (Figures 1d, b and 8a); similarly, another report showed that TNF-α, IL-1β, and IL-6 have consistently been detected in pSS minor salivary gland biopsy and conjunctiva samples [29]. In addition, the expression of IL-17/IL-23 in salivary glands tended to be higher in patients with SS with higher biopsy focus score. However, in that report, like a previous report [23], there was no direct correlation between the IL-17/IL-23 expressions and clinical profiles such as the degree of abnormal salivary flows, the presence of antinuclear antibody, rheumatoid factor, anti-Ro, anti-La, and extraglandular involvements (data not shown).

The biological relevance of our data requires comment. We examined the pathophysiologic mechanism of the TLR/IL-17 pathway in patients with SS by using PBMCs. Thus, we failed to clarify the role of epithelial cells, which are thought to be central players in the pathogenesis of pSS [40,41]. However, although the ductal epithelial as well as the infiltrating mononuclear cells expressed TLRs in our results and previous reports [13,14], IL-17, the main effector cytokine in Th17 cells, is produced mainly by activated CD4^+ ^T cells. In this study, we also demonstrated that the major sources of IL-17 are CD4^+ ^T cells by using PBMCs (Figure 2a). Ductal epithelial cells were variably positive for IL-17 stain, whereas IL-17 was highly expressed mainly in the infiltrating CD4^+ ^T cells in the salivary glands of patients with SS (Figure 2b). Therefore, it seems that our data using PBMCs are biologically relevant, although we did not completely rule out IL-17 production by ductal epithelial cells.

## Conclusions

The present study demonstrated that TLR2 ligation induces the production of IL-23/IL-17 via IL-6, STAT3, and NF-κB pathways, and so we have determined the role of the TLR/IL-17 pathway in the pathophysiology of SS for the first time. Therefore, our data underline the importance of the TLR/IL-17 pathway as a strong potential candidate for the therapeutic modulation of SS.

## Abbreviations

DC: dendritic cell; ELISA: enzyme-linked immunosorbent assay; ESR: erythrocyte sedimentation rate; IL: interleukin; mAb: monoclonal antibody; NF-κB: nuclear factor-kappa-B; PAMP: pathogen-associated molecular pattern; PBMC: peripheral blood mononuclear cell; PCR: polymerase chain reaction; pSS: primary Sjögren's syndrome; RA: rheumatoid arthritis; SLE: systemic lupus erythematosus; SS: Sjögren's syndrome; STAT: signal transducer and activator of transcription; TGF-β: transforming growth factor-beta; Th: T helper; TLR: Toll-like receptor; TNF-α: tumor necrosis factor-alpha.

## Competing interests

The authors declare that they have no competing interests.

## Authors' contributions

S-KK and M-LC performed *in vitro *experiments and drafted the manuscript. Y-MH and H-JO performed immunohistochemical and confocal staining. M-KP and S-YL carried out flow cytometry analysis. YJW assisted in *in vitro *experiments. JHJ and K-SP assisted in drafting the manuscript. H-YK assisted in designing the study. S-HP conceived the study and drafted and edited the manuscript. All authors read and approved the final manuscript.
